# Consequences of high temperatures and premature mortality on the transcriptome and blood physiology of wild adult sockeye salmon (*Oncorhynchus nerka*)

**DOI:** 10.1002/ece3.274

**Published:** 2012-07

**Authors:** Ken M Jeffries, Scott G Hinch, Thomas Sierocinski, Timothy D Clark, Erika J Eliason, Michael R Donaldson, Shaorong Li, Paul Pavlidis, Kristi M Miller

**Affiliations:** 1 Centre for Applied Conservation Research and Department of Forest Sciences, University of British Columbia2424 Main Mall, Vancouver, BC, V6T 1Z4, Canada; 2 Department of Psychiatry, Centre for High-Throughput Biology, University of British Columbia2125 East Mall, Vancouver, BC, V6T 1Z4, Canada; 3 Australian Institute of Marine SciencePMB 3, Townsville MC, Townsville, QLD 4810, Australia; 4 Department of Zoology, University of British Columbia6270 University Blvd., Vancouver, BC, V6T 1Z4, Canada; 5 Fisheries and Oceans Canada, Molecular Genetics SectionPacific Biological Station, 3190 Hammond Bay Road, Nanaimo, BC, V9T 6N7, Canada

**Keywords:** Ecological genomics, Pacific salmon, premature mortality, spawning migration, stress, temperature

## Abstract

Elevated river water temperature in the Fraser River, British Columbia, Canada, has been associated with enhanced mortality of adult sockeye salmon (*Oncorhynchus nerka*) during their upriver migration to spawning grounds. We undertook a study to assess the effects of elevated water temperatures on the gill transcriptome and blood plasma variables in wild-caught sockeye salmon. Naturally migrating sockeye salmon returning to the Fraser River were collected and held at ecologically relevant temperatures of 14°C and 19°C for seven days, a period representing a significant portion of their upstream migration. After seven days, sockeye salmon held at 19°C stimulated heat shock response genes as well as many genes associated with an immune response when compared with fish held at 14°C. Additionally, fish at 19°C had elevated plasma chloride and lactate, suggestive of a disturbance in osmoregulatory homeostasis and a stress response detectable in the blood plasma. Fish that died prematurely over the course of the holding study were compared with time-matched surviving fish; the former fish were characterized by an upregulation of several transcription factors associated with apoptosis and downregulation of genes involved in immune function and antioxidant activity. Ornithine decarboxylase (ODC1) was the most significantly upregulated gene in dying salmon, which suggests an association with cellular apoptosis. We hypothesize that the observed decrease in plasma ions and increases in plasma cortisol that occur in dying fish may be linked to the increase in ODC1. By highlighting these underlying physiological mechanisms, this study enhances our understanding of the processes involved in premature mortality and temperature stress in Pacific salmon during migration to spawning grounds.

## Introduction

**Figure 1:**
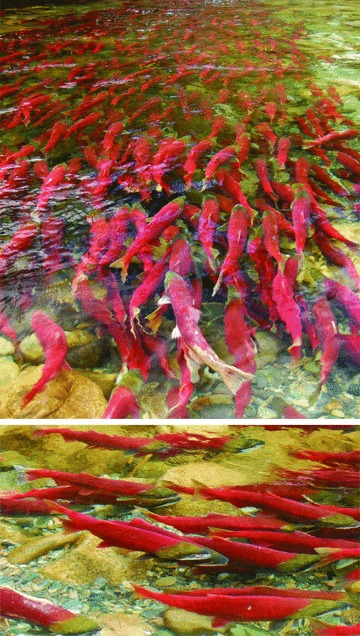
Adult Fraser River sockeye salmon (*Oncorhynchus nerka*) at their natal freshwater spawning grounds. Photos: M. T. Casselman.

As a consequence of climate change, many populations of sockeye salmon (*Oncorhynchus nerka*; Fig. 1) now experience significantly warmer river conditions during their once-in-a-lifetime spawning migration from the Pacific Ocean to freshwater spawning grounds. Spawning migrations during warm water periods are associated with increased levels of en route mortality (premature mortality during migration) and prespawn mortality (premature mortality on spawning grounds) in sockeye salmon (Gilhousen 1990; Macdonald et al. 2010; reviewed in Hinch and Martins 2011). Unless sockeye salmon are able to adapt to these climatic shifts, continued river warming will likely result in a reduced number of individuals reaching spawning grounds in the future (Hague et al. 2011; Martins et al. 2011). To assist with understanding the pervasive role of temperature in determining spawning success, our aim herein is to identify some of the physio-logical and molecular mechanisms involved in a temperature-induced stress response in sockeye salmon.

Interpretations of temperature-induced physiological changes during spawning migrations are complicated by the fact that sockeye salmon are simultaneously maturing, senescing, and starving (having ceased feeding prior to river entry). The fish also undergo dramatic physiological and morphological restructuring in preparation for spawning (Groot and Margolis 1991). This is followed by a period of rapid senescence, which typically occurs within days postspawning and is accompanied by immunosuppression and organ deterioration (Dickhoff 1989; Finch 1990). The exact factors that cause postspawning mortality remain contentious, but may include parasites and disease (Servizi and Jensen 1977; Gilhousen 1990; Bradford et al. 2010) and reduced osmoregulatory ability (Shrimpton et al. 2005; Jeffries et al. 2011). Currently, it is unclear whether premature mortality associated with chronically elevated river temperatures results from acceleration of natural senescence processes, elevated virulence of disease, cardiovascular impairment, or some other factor. However, as yet, the molecular mechanisms involved in premature mortality in Pacific salmon have not been examined experimentally.

The development of a 16K gene salmonid cDNA micro-array through the GRASP project (von Schalburg et al. 2005) has facilitated a rapid growth in salmonid genomics research. This salmonid microarray has been used to determine genomic responses to a variety of infectious diseases negatively affecting the aquaculture industry, including infectious hematopoietic necrosis (IHN) virus (Miller et al. 2007), amoebic gill disease (Young et al. 2008), and saprolegniasis infection (Roberge et al. 2007). This microarray has also been used to characterize the heat stress response in rainbow trout (Lewis et al. 2010) and killifish (Healy et al. 2010). It has been applied to wild fish to examine molecular processes associated with intricate reproductive behaviors (Aubin-Horth et al. 2005), and to understand complex physiological processes in wild-caught adult sockeye salmon during spawning migrations (Miller et al. 2009, 2011; Evans et al. 2011), demonstrating the utility of this tool in understanding pertinent aspects of the biology of wild salmon.

Here, we utilize the GRASP 16K gene salmonid cDNA microarray to examine, for the first time, the physiological and molecular mechanisms associated with temperature stress and premature mortality in wild-caught adult sockeye salmon from two populations held under controlled conditions. We compare gene expression profiles of sockeye salmon held at ecologically relevant migration temperatures (14°C and 19°C) to determine the common effect of elevated water temperature on cellular processes between populations in non-lethally sampled gill tissue. We also contrast gene expression profiles of moribund fish with time-matched surviving fish to examine cellular processes associated with premature mortality. The gene expression profiles are complemented by blood plasma analyses to provide the most comprehensive examination to date of the mechanisms underlying temperature stress and premature mortality in adult sockeye salmon.

## Materials and Methods

### Fish collection and thermal holding

Adult sockeye salmon (*n* = 130) from multiple populations were collected by beach seine from the Fraser River, British Columbia, Canada, 5–7 September 2007. Water temperatures during collection ranged from 15°C to 17°C. Fish were transported 45 min by road in aerated approximately 12°C water to the Fisheries and Oceans Canada Cultus Lake Laboratory, near Chilliwack, British Columbia, Canada, where they were randomly distributed among four 8000-L aerated tanks at 12°C and at equal fish densities (sand-filtered and ultraviolet-sterilized water). Each tank contained a submersible pump that created a water flow of approximately 0.3 m s^−1^ into which the fish were able to orient and maintain position by continuous, gentle swimming. Fish were given three to six days to recover from transport, at which time all fish appeared vigorous and there were no external signs of disease. The tank water temperatures were subsequently raised at a rate of 2–2.5°C day^−1^ until the test temperatures of 14°C and 19°C were reached (two tanks at each temperature).

### Tissue collection

DNA identification (Beacham et al. 2005) confirmed that the sockeye salmon sampled for blood and gill were from the Lower Adams and Chilko River populations. Any other populations collected were not used in the present study. After seven days of a stable test temperature (termed “experimental day 7”), gill tissue was nonlethally sampled from surviving fish in both temperature groups (termed “survivor” samples; 19°C treatment: *n* = 3 Lower Adams, *n* = 8 Chilko River; 14°C treatment: *n* = 6 Chilko River) to determine the effect of water temperature on gene expression. A time point of seven days was chosen because it approximately represents the period of time that these populations spend moving through the Lower Fraser River and the Fraser River Canyon (English et al. 2005); the sections of the Fraser River where these populations often experience the highest water temperatures. Fish were considered “survivors” if they were swimming and maintaining position in the current on experimental day 7. There was higher mortality in the 19°C treatment (50% mortality after seven days compared with 25% mortality after seven days at 14°C). Survival patterns were continuously monitored in these fish for an additional 13 days. Fish that no longer maintained equilibrium around experimental day 7 (mean of 6.26 ± 0.46 days), but were still ventilating, were removed from the tanks, sampled for gill tissue and sacrificed (termed “moribund” samples; 19°C treatment: *n* = 5 Lower Adams, *n* = 8 Chilko River; 14°C treatment: *n* = 4 Lower Adams, *n* = 6 Chilko River). Individuals from the survivor and moribund groups were mutually exclusive. Gill samples were immediately flash frozen in liquid nitrogen and stored at –80°C until analysis.

Blood samples obtained from the caudal vasculature using a heparinized vacutainer were collected at the same time as the gill samples for survivors and moribund fish to detect changes in osmoregulatory and stress indices. Blood samples (∼3 mL) were immediately centrifuged for 7 min at 7000 ×*g* and plasma was stored at –80°C prior to analyses. Plasma variables were measured using the procedures outlined in Farrell et al. (2001).

### RNA extraction, amplification, labeling, and hybridization

The prehybridization protocols used in the present study were similar to those used in Miller et al. (2009, 2011). Briefly, total RNA from gill samples was purified from individual fish gill using Magmax™-96 for Microarrays Kits (Ambion Inc., Austin, TX) with a Biomek FXP (Beckman-Coulter, Mississauga, ON, Canada) automated liquid-handling instrument. Approximately 0.5 mg of gill tissue per fish was homogenized with stainless steel beads in 400 µL of TRI-reagent (Ambion Inc.) on a MM301 mixer mill (Retsch Inc., Newtown, PA). Aliquots of 100 µL of homogenate were pipetted into 96-well plates and extractions were carried out using the “no-spin procedure” according to the manufacturer's instructions. In the final step, RNA was eluted and RNA yield was determined by measuring the A260. Purity was assessed by measuring the A260/A280 ratio. Solutions of RNA were stored at –80°C until used for the microarray experiment or for quantitative real-time PCR (qRT-PCR).

Total RNA (500 ng–5 µg) was amplified using a MessageAmpTMII-96 kit (Ambion Inc.) according to manufacturer's instructions. Five micrograms of aRNA were reverse transcribed into cDNA, purified using Zymo-25 Clean-Up columns (Zymo Research, Irvine, CA) and labeled with Alexa dyes using the Invitrogen Indirect Labelling Kit (Invitrogen, Carlsbad, CA) following manufacturer's instructions. Treatment samples were labeled with Alexa 555 and reference samples, comprised of a pool of RNA from all the fish used in the study, were labeled with Alexa 647. After 1 h at room temperature, 50 µL of DNA binding buffer was added to each Alexa tube and sample and references for each slide were combined and purified in Zymo-25 Clean-Up columns (Zymo Research). Labeled cDNA was washed three times with DNA wash buffer (Zymo Research) and eluted in 9 µL of 1× TE buffer. Two microliters of poly dA were added to the labeled cDNA, followed by 10-min denaturation at 80°C and the addition of 125 µL of prewarmed (65°C) SlideHybe3 buffer (Ambion Inc.). Samples were loaded into hybridization chambers in a Tecan-HS4800 Pro Hybridization Station (Tecan Trading AG, Männedorf, Switzerland).

### Hybridization, normalization, and quality control

Each individual in the array study was hybridized on a single slide against a reference control. All steps from washing, hybridization, denaturation, and slide drying were carried out automatically on the Tecan-HS4800 Pro Hybridization Station. Fluorescent images were scanned using a Perkin Elmer ScanArray Express (Perkin Elmer, Boston, MA) and the signal-to-noise ratio was adjusted for optimized visualization of each image. The images were quantified using the program Imagene (BioDiscovery, El Segundo, CA). Each slide was normalized in BASE using the print-tip LOESS method. A detailed description of how slide quality and outliers were assessed can be found in supplemental material from Miller et al. (2011). The microarray data have been deposited in the Gene Expression Omnibus (http://www.ncbi.nlm.nih.gov/geo/) with the accession number GSE33586.

### Quantitative RT-PCR

Five genes were analyzed using qRT-PCR to validate micro-array results associated with a temperature response or with premature mortality (Table 1). Primers for four of the genes were designed in-house, with primers developed to equally match contigs of rainbow trout and Atlantic salmon. Transcription factor Jun B (JUNB) primers were published in Momoda et al. (2007). cDNA was synthesized from total RNA (1 µg) using an iScriptTM cDNA Synthesis Kit (Bio-Rad Laboratories, Inc., Hercules, CA) following manufacturer's instructions. A 1:2.5 dilution of cDNA was used as a template in the qRT-PCR assays. The relative quantification (RQ) assays were performed on an ABI 7900HT Fast real-time PCR system (Applied Biosystems, Carlsbad, CA) in 384-well plates using 20 µL reaction volumes that included 10 µL Kapa SYBR fast QPCR Master Mix (2×) (Kapa Biosystems, Inc., Woburn, MA), 0.4 µL of a mixture of 0.2 µM forward and reverse primers, 2 µL of diluted cDNA, and 7.6 µL of RNase/DNase-free water. The cycling conditions were 95°C for 3 min followed by 40 cycles of 95°C for 3 sec and 60°C for 30 sec, and a dissociation stage was added at each RQ run to ensure the presence of only a single amplicon. All samples were run in duplicate and with nontemplate controls included. Relative expression of target genes was determined using the comparative Ct method (Livak and Schmittgen 2001; Schmittgen and Livak 2008). Target gene expression was normalized to two housekeeping genes (78d16.1 and BMP4); these housekeeping genes were not responsive to the experimental factors (M. R. Donaldson and S. Li, unpubl. data).

**Table 1:** Primer sequences and expressed sequence tag (EST) numbers for the genes selected for quantitative real-time PCR (qRT-PCR) analysis

Gene group	Gene name	Gene symbol	EST number	Primers (5′-3′)
Temperature responsive	Heat shock protein 90	HSP90AB1	CB493960	F-TGGGCTACATGGCTGCCAAG
				R-TCCAAGGTGAACCCAGAGGAC
	Serpin peptidase inhibitor	SERPINH1	CA063723	F-TCCACTTTCCACCCTGCAAAG
				R-AGTTTGGTTGGCAAATGGCATAG
	Cold-inducible RNA binding protein	CIRBP	CB499204	F-AAGCTGTGATTGTGCTCTAAAGAC
			CA048095	R-TCCCACTTAGCATTCCATCCTTG
			CA064457	
Mortality responsive	Cytochrome c	cyt c	CB494539	F-CGAGCGTGCAGATCTTATAGC
				R-CTTCTCCGCTGAACAGTTGATG
	Transcription factor Jun B	JUNB	N/A^1^	F-CTACACGCACAGCGATATTCG
				R-TCGTCGCTGCTCTGCATGT
Housekeeping genes	Bone morphogenetic protein 4	BMP4	CA056395	F-TTGCCCATAGTCAGTGTTAGCG
				R-GTGCCATCTCCATGCTCTACC
	Si:dkey-78d16.1 protein [*Danio rerio*]	N/A	CA056739	F-AAAGGTCCCACGCTCCAAAC
				R-ACACACCCATCTGTCTCATCACC

1 Primer sequences for JUNB were previously published in Momoda et al. (2007).

### Data analysis

We utilized both supervised and unsupervised analysis approaches on the microarray data. Unsupervised principal component analysis (PCA), conducted to identify the major transcriptional trajectories in the data, was computed using R as detailed in Alter et al. (2000). Imputed values were used when values were missing for the PCA. This method provides a ranking of probes characterizing their contribution (in terms of variance) with respect to the principal component of interest (i.e., the PC loading). Both positive and negative loadings were considered. The correlation of each PC axis with the blood plasma variables (continuous variables) was assessed using Spearman rank correlations. The relationship between each PC and the treatment groups (binary categorical variables) was assessed using Mann–Whitney U (MWU) tests. Supervised analyses were used to compare between treatment groups using MWU tests. Genes were considered significantly different between treatment groups at *q* < 0.05 (the false discovery rate [FDR] corrected *P*-value). However, genes significant at *P* < 0.001, consistent with previous work on sockeye salmon (Miller et al. 2009), were also considered. It is important to note that multiple contigs of the same gene may reflect different expressed sequence tags (ESTs) of the same gene or duplicated copies of that gene; hence, we included all ESTs for each gene that was significant for our analyses.

Functional analysis was performed with ErmineJ (Lee et al. 2005) using the receiver operator characteristic (ROC) scoring method. The ROC method is a nonthreshold method performed on all gene rankings, which may be more robust than using raw gene scores (Lee et al. 2005). All three categories of the GO hierarchy (Biological Process, Molecular Function, and Cellular Component) were considered, limited to groups with 5–100 genes. However, only the categories Biological Process and Molecular Function are presented. The “best-scoring replicate” method was used in ErmineJ to handle repeated measurements of the same gene. Gene sets in the ErmineJ analyses were considered significant at Benjamini–Hochberg FDR < 0.1 (e.g., Lockwood and Somero 2011).

All statistical tests on qRT-PCR and blood data were performed using SAS software version 9.1 (SAS Institute Inc., Cary, NC) or SigmaPlot version 11.0 (SYSTAT Software, Inc., Chicago, IL). Differences in blood properties between temperature treatments or survival versus moribund status were assessed using *t*-tests with a Bonferroni adjusted critical alpha of 0.016 (Zar 1999) for the blood osmoregulatory (plasma osmolality, chloride, and sodium) and stress (plasma glucose, lactate, and potassium) indices. Potassium was included as a stress variable on the basis that it increases concurrently with lactate in stressed and moribund Pacific salmon (Jeffries et al. 2011). If the assumption of normality could not be met as determined by Kolmogorov–Smirnov tests, non-parametric MWU tests were used (Sokal and Rohlf 1995). Statistical differences in qRT-PCR results were analyzed by two-factor analyses of variance (ANOVAs) with temperature and survival as factors. Tukey-Kramer pairwise comparisons were made a posteriori using the Bonferroni adjustment method with an adjusted critical alpha of 0.01 for the five genes used for qRT-PCR analysis. For the two-factor ANOVAs, homogeneity of variances was assessed by Bartlett's tests and normality was tested using Kolmogorov–Smirnov tests. All data were log_10_-transformed if the assumption of homogeneity of variances could not be met.

## Results

### Temperature effects on survivors

**Figure 2:**
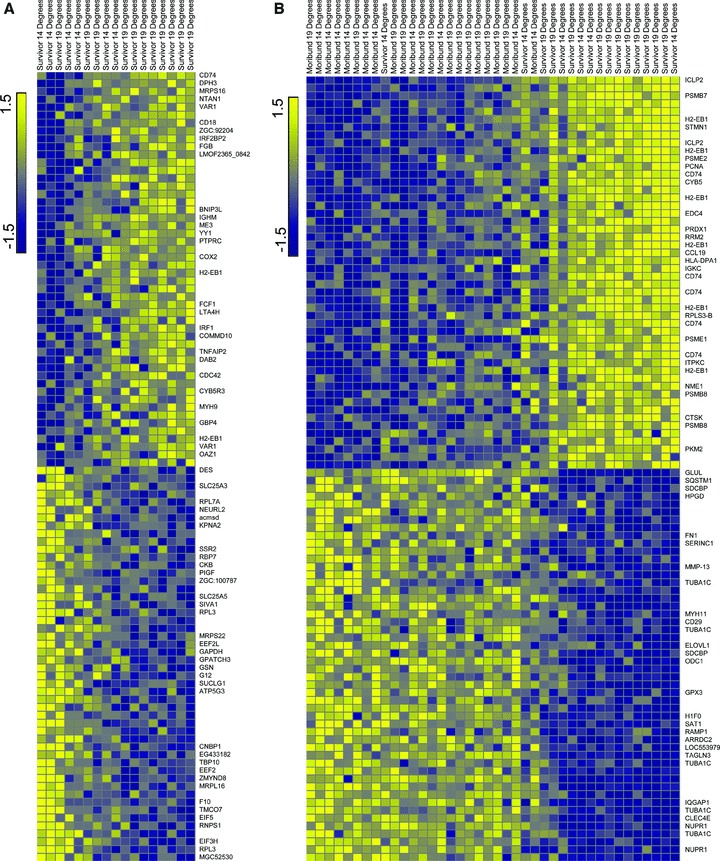
Heat maps showing the top 50 positively and top 50 negatively loaded genes for the principal component (PC) most related with (A) temperature in survivors (PC3) and (B) for comparisons between survivors and fish that became moribund (PC1). Relative expression levels are indicated by the color scale with yellow indicating upregulated and blue indicating downregulated. Ordering of fish reflects their relative PC scores along the PC axis. Gene symbols (if available) are presented along the right side of the heat map.

The temperature treatments were most strongly associated with PC3 (MWU, *P* < 0.02) in fish from the survivors group. Fish in the 19°C treatment were generally on the positive end of the PC3 axis (Fig. 2A). Interestingly, the three 19°C treatment fish that were grouped on the negative end of the PC3 axis, with the 14°C fish, died one to two days after the experimental day 7 sampling. ErmineJ ROC analysis indicated that functional categories involved in protein biosynthesis, oxidative phosphorylation, immune response, and protein targeting and transport were significantly affected by the temperature treatments (Table 2). Plasma chloride (ρ = 0.74), sodium (ρ = 0.57), and osmolality (ρ = 0.57) were positively correlated with PC3 and therefore were generally higher in individuals in the 19°C treatment. In all cases, the population from which each fish belonged (Lower Adams or Chilko River) was not significantly associated with any of the PCs (MWU, *P* > 0.05); hence, populations were pooled for all the analyses. However, as there were only three surviving Lower Adams fish, the power to detect differences among populations was low. Pooling the populations for subsequent analyses allowed for the detection of a more general response to temperature that is common between these populations.

**Table 2:** Functional analysis performed using the receiver operator characteristic (ROC) scoring method in ErmineJ. The GO categories Biological Process and Molecular Function are presented here. Only gene sets significant at false discovery rate corrected *P*-values < 0.1 are presented

GO category	GO ID	Name	GO ID	Name	# of genes	Raw score	Corrected *P*-value
**Temperature effects on survivors**							
Biological process	GO:0065007	Biological regulation	GO:0006446	Regulation of translational initiation	14	0.81	0.0087
			GO:0006417	Regulation of translation	14	0.79	0.011
			GO:0010608	Posttranscriptional regulation of gene expression	18	0.74	0.015
			GO:0008361	Regulation of cell size	14	0.72	0.065
	GO:0044237	Cellular metabolic process	GO:0006119	Oxidative phosphorylation	8	0.86	0.011
			GO:0006091	Generation of precursor metabolites and energy	19	0.74	0.016
	GO:0009058	Biosynthetic process	GO:0006412	Translation	44	0.65	0.020
			GO:0006633	Fatty acid biosynthetic process	6	0.85	0.039
			GO:0006487	Protein amino acid N-linked glycosylation	12	0.75	0.045
	GO:0002376	Immune system process	GO:0006955	Immune response	37	0.66	0.028
	GO:0009987	Cellular process	GO:0006886	Intracellular protein transport	45	0.63	0.045
			GO:0006605	Protein targeting	20	0.69	0.049
			GO:0016197	Endosome transport	7	0.81	0.054
			GO:0007005	Mitochondrion organization	13	0.72	0.090
			GO:0006839	Mitochondrial transport	7	0.79	0.089
			GO:0034613	Cellular protein localization	40	0.62	0.098
	GO:0050896	Response to stimulus	GO:0006952	Defense response	38	0.64	0.063
			GO:0006954	Inflammatory response	21	0.67	0.088
	GO:0043170	Macromolecule metabolic process	GO:0009100	Glycoprotein metabolic process	19	0.68	0.081
	GO:0055114	Oxidation-reduction process	GO:0045333	Cellular respiration	5	0.84	0.090
Molecular function	GO:0005488	Binding	GO:0003723	RNA binding	69	0.63	0.011
			GO:0008135	Translation factor activity, nucleic acid binding	26	0.67	0.046
			GO:0008022	Protein C-terminus binding	14	0.70	0.096
	GO:0003824	Catalytic activity	GO:0016651	Oxidoreductase activity, acting on NADH or NADPH	14	0.76	0.020
			GO:0016655	Oxidoreductase activity, acting on NADH or NADPH, quinone or similar compound as acceptor	8	0.78	0.084
			GO:0008137	NADH dehydrogenase (ubiquinone) activity	8	0.76	0.095
	GO:0005198	Structural molecule activity	GO:0003735	Structural constituent of ribosome	27	0.68	0.025
			GO:0005198	Structural molecule activity	53	0.61	0.080
**Temperature effects on moribund fish**							
Biological process	GO:0065007	Biological regulation	GO:0006355	Regulation of transcription, DNA-dependent	98	0.61	0.030
			GO:0051252	Regulation of RNA metabolic process	97	0.60	0.040
			GO:0051130	Positive regulation of cellular component organization	7	0.83	0.074
			GO:0006446	Regulation of translational initiation	14	0.73	0.072
			GO:0051246	Regulation of protein metabolic process	36	0.64	0.091
			GO:0009892	Negative regulation of metabolic process	58	0.61	0.092
	GO:0009058	Biosynthetic process	GO:0006412	Translation	46	0.64	0.043
	GO:0051179	Localization	GO:0015031	Protein transport	47	0.63	0.068
			GO:0008104	Protein localization	54	0.61	0.088
	GO:0009057	Catabolic process	GO:0009057	Macromolecule catabolic process	50	0.62	0.080
	GO:0009987	Cellular process	GO:0051649	Establishment of localization in cell	98	0.58	0.087
Molecular function	GO:0005198	Structural molecule activity	GO:0003735	Structural constituent of ribosome	28	0.70	0.039
			GO:0005198	Structural molecule activity	56	0.62	0.074
			GO:0005200	Structural constituent of cytoskeleton	13	0.73	0.083
	GO:0051082	Binding	GO:0051082	Unfolded protein binding	14	0.72	0.081
**Moribund vs. survivor patterns**							
Biological process	GO:0009058	Biosynthetic process	GO:0006412	Translation	46	0.68	0.0027
	GO:0006950	Response to stress	GO:0009611	Response to wounding	40	0.65	0.042
			GO:0042221	Response to chemical stimulus	50	0.62	0.094
			GO:0006952	Defense response	41	0.63	0.091
	GO:0009987	Cellular process	GO:0016192	Vesicle-mediated transport	57	0.61	0.091
			GO:0006928	Cellular component movement	33	0.65	0.086
			GO:0046907	Intracellular transport	87	0.59	0.083
	GO:0044237	Cellular metabolic process	GO:0006690	Icosanoid metabolic process	6	0.82	0.091
	GO:0048731	System development	GO:0048513	Organ development	100	0.58	0.092
			GO:0007399	Nervous system development	75	0.59	0.097
	GO:0051179	Localization	GO:0051674	Localization of cell	32	0.64	0.098
Molecular function	GO:0005198	Structural molecule activity	GO:0003735	Structural constituent of ribosome	28	0.73	0.0050
			GO:0005198	Structural molecule activity	56	0.62	0.050
	GO:0005488	Binding	GO:0003723	RNA binding	72	0.62	0.024
			GO:0003746	Translation elongation factor activity	5	0.85	0.091
			GO:0005543	Phospholipid binding	16	0.70	0.096
	GO:0016209	Antioxidant activity	GO:0016209	Antioxidant activity	7	0.84	0.046
	GO:0003824	Catalytic activity	GO:0008238	Exopeptidase activity	7	0.81	0.087
			GO:0070011	Peptidase activity, acting on L-amino acid peptides	33	0.65	0.093
			GO:0008233	Peptidase activity	35	0.64	0.090
	GO:0005215	Transporter activity	GO:0008565	Protein transporter activity	13	0.72	0.096

**Figure 3:**
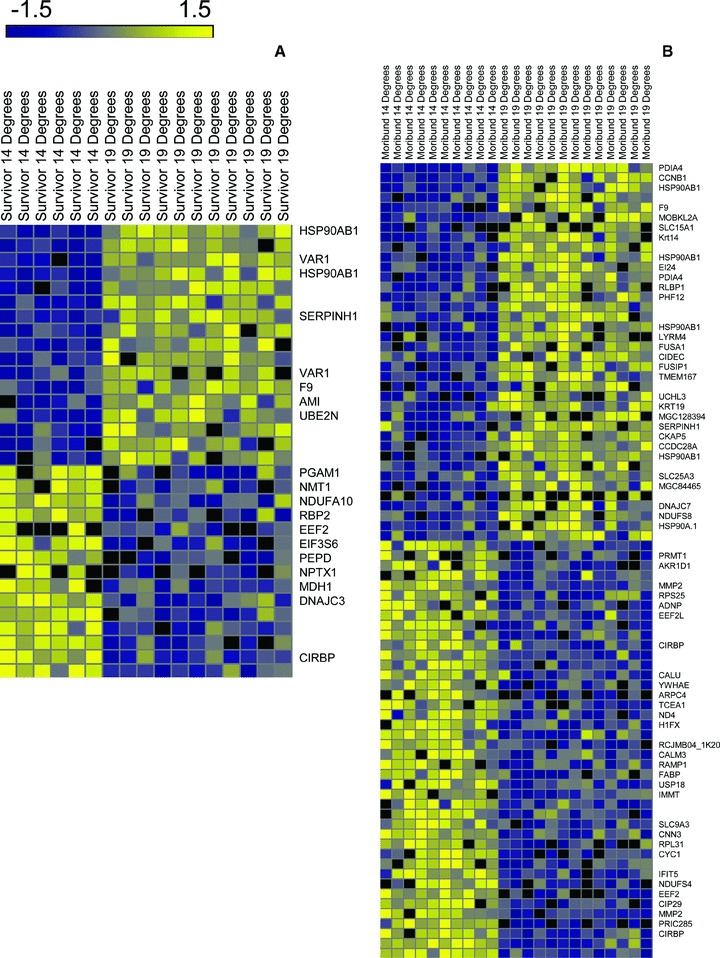
Heat maps showing the genes significantly different between temperature treatments for (A) survivors (32 genes total, 30 unique genes) and (B) moribund (80 genes total, 74 unique genes) sockeye salmon at *P* < 0.001 (Mann–Whitney U tests) when fish were grouped based on treatment. Relative expression levels are indicated by the color scale with yellow indicating upregulated and blue indicating downregulated. Missing values are shown in black. Gene symbols (if available) are presented along the right side of the heat map.

**Figure 4:**
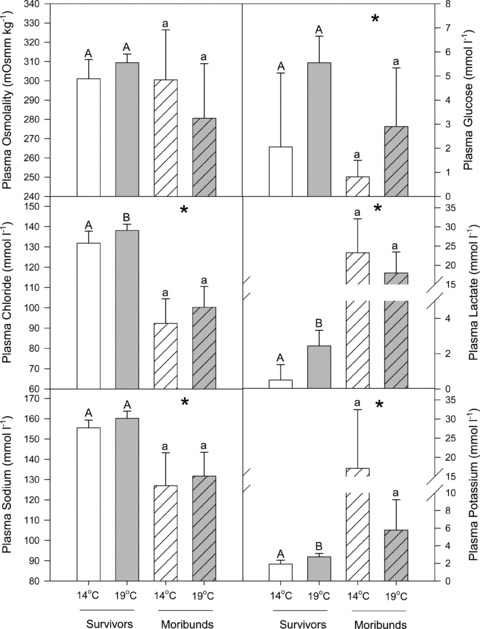
Blood plasma properties for survivors (open bars) and moribund (hatched bars) sockeye salmon held at 14°C and 19°C (white and gray bars, respectively). Capital letters indicate statistical differences between temperature treatments for survivors; lower case letters indicate statistical differences between temperature treatments for moribund fish. Statistical differences between moribund fish and survivors are indicated by (*).

Relatively few genes were significantly different between the temperature treatment groups among survivors when supervised approaches were used, potentially due to small sample sizes. Of the 32 genes that had different levels of expression at *P* < 0.001 (MWU tests; Fig. 3A), only one gene was significantly upregulated in the 19°C treatment at *q <* 0.05 (heat shock protein 90; HSP90AB1). However, several of the 32 genes are known to be temperature responsive (e.g., HSP90AB1, serpin peptidase inhibitor [SERPINH1; also called heat shock protein 47], and cold-inducible RNA-binding protein [CIRBP]) or are biologically relevant (see Discussion). Surprisingly, only two of the 32 genes (mitochondrial ribosomal protein [VAR1] and eukaryotic translation elongation factor 2 (EEF2]) were highly loaded on the PC3 axis. Moreover, as observed with PC3, survivors held at 19°C had higher plasma chloride (*t*-test, *P* = 0.014), but also possessed higher plasma lactate (*t*-test, *P* < 0.001) and potassium (*t*-test, *P* < 0.005) than survivors held at 14°C (Fig. 4). Survivors held at 19°C had higher plasma osmolality (*t*-test, *P* = 0.037) and glucose (MWU, *P* = 0.039) than survivors held at 14°C; however, they were not significantly different after Bonferroni correction.

### Temperature effects on moribund fish

**Figure 5:**
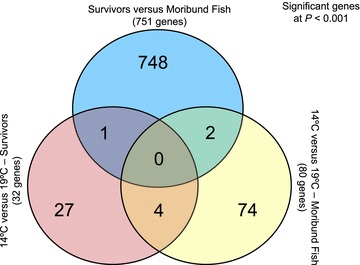
Venn diagram of significant genes from the three main supervised comparisons in the present study (effects of the temperature treatments on survivors and moribund fish, and the effects of survival status on gene expression). Numbers represent the number of differentially expressed genes at *P* < 0.001 as determined by Mann–Whitney U tests.

The temperature treatment was nearly significantly associated with PC1 (MWU, *P* = 0.06) and marginally significantly associated with PC2 (MWU, *P* = 0.049) in the fish that became moribund. Only plasma glucose was strongly correlated with PC1 (ρ = 0.81). ErmineJ ROC analysis on PC2 indicated that functional categories associated with protein biosynthesis, cell structural components, and protein transport and binding were significantly affected by the temperature treatments in fish that became moribund (Table 2). When the moribund fish were grouped by temperature treatment and directly contrasted, 80 genes had different levels of expression at *P* < 0.001 (MWU tests; Fig. 3B) with nine genes differentially expressed at *q* < 0.05 (Upregulated at 19°C: protein disulfide isomerase family A, member 4 [PDIA4], cyclin B1 [CCNB1], HSP90AB1; Downregulated at 19°C: hyperosmotic glycine rich protein and splicing factor, arginine/serine-rich 2 [no gene symbols available], CIRBP, peroxisomal proliferator-activated receptor A interacting complex 285 [PRIC285], matrix metallopeptidase 2 [MMP2], and cytokine-induced protein 29 KDA [CIP29]). The genes F9 (coagulation factor IX), SERPINH1, CIRBP and splicing factor, arginine/serine-rich 2 (Fig. 5), and copies of HSP90AB1 and EEF2 (different contigs between the two groups) had the same transcriptional response due to the temperature treatments as fish in the survivors group. Moribund fish held at 19°C had higher plasma glucose (MWU, *P* = 0.021) and lower plasma potassium (MWU, *P* = 0.039) than moribund fish held at 14°C, but they were not significantly different after Bonferroni correction (Fig. 4).

### Moribund versus survivor patterns

**Figure 6:**
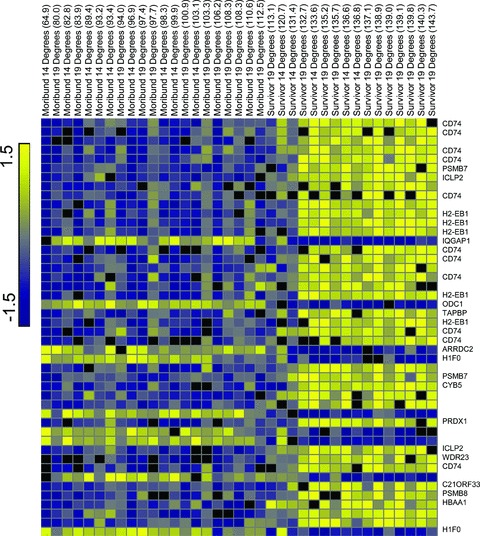
Heat map showing the genes most significantly correlated with plasma chloride concentration (mmol l^-1^, in parentheses) at *P* < 1.0 × 10^−7^ in survivors and moribund sockeye salmon. Relative expression levels are indicated by the color scale with yellow indicating upregulated and blue indicating downregulated. Missing values are shown in black. Gene symbols (if available) are presented along the right side of the heat map.

When all samples were combined into a single PCA, PC1 was strongly associated with differences between fish that survived versus those that became moribund (MWU, *P* < 1 × 10^−7^), but did not differentiate fish on the basis of temperature (MWU, *P* > 0.05). In fact, temperature was not significantly associated with the first five PCs, which explained much (25.9%) of the variance in the data. Survivors were generally located on the positive end of the PC1 axis (Fig. 2B). The two survivor fish that were grouped with the moribund fish died one to two days after the experimental day 7 sampling. ErmineJ ROC analysis indicated that functional categories involved in immune response, protein biosynthesis, antioxidant activity, and protein catalytic activity were significantly affected in fish that became moribund (Table 2). Plasma chloride (ρ = 0.75), sodium (ρ = 0.70), and glucose (ρ = 0.67) were positively correlated with PC1 (and therefore survivorship), while plasma lactate (ρ = –0.75) was negatively correlated with PC1. Because plasma chloride levels are the strongest predictor of longevity in adult sockeye salmon (Jeffries et al. 2011) and were highly correlated with PC1, we assessed the 46 genes most correlated (*P* < 1 × 10^−7^) with plasma chloride levels (Fig. 6). Many contigs of the immune response genes, MHC class II associated invariant chain (CD74), histocompatibility complex class II (H2-EB1, also called MHC II), and invariant chain-like protein 2 (ICLP2) along with ornithine decarboxylase 1 (ODC1), were among the 46 genes most correlated with plasma chloride levels.

**Figure 7:**
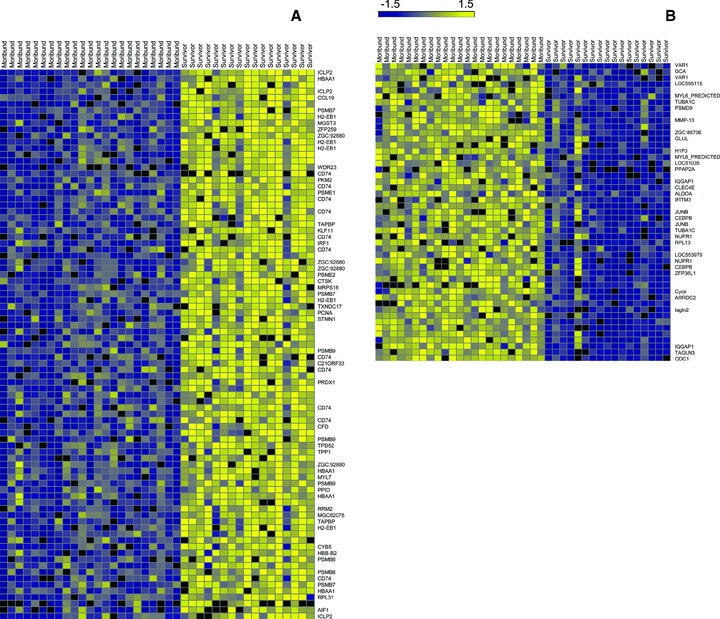
Heat maps showing the genes most significantly (A) upregulated (87 genes total, 59 unique genes) and (B) downregulated (49 genes total, 42 unique genes) in survivors at *P* < 1.0 × 10^−6^ (Mann–Whitney U tests) when sockeye salmon were grouped based on survival. Relative expression levels are indicated by the color scale with yellow indicating upregulated and blue indicating downregulated. Missing values are shown in black. Gene symbols (if available) are presented along the right side of the heat map.

Because the temperature treatments were not a significant factor in the PCA when survivors and moribund fish were pooled for analysis, both temperature treatment groups were pooled for the subsequent analysis. When survivors and moribund fish were contrasted directly, 751 genes had different levels of expression at *P* < 0.001 (MWU tests), with 1503 genes differentially expressed at *q* < 0.05 (Fig. 7). There was little overlap between these 751 genes and the significant gene lists associated with the temperature treatments (Fig. 5). The most significantly upregulated gene in the moribund fish was ODC1 (MWU, *P* < 1.0 × 10^−13^). Of the 136 genes that had different levels of expression at *P* < 1.0 × 10^−6^, there was significant overlap in the transcriptional response with the genes most correlated with the PC1 axis (Fig. 2B). Survivors had significantly higher plasma chloride (MWU, *P* < 0.001), sodium (MWU, *P* < 0.001), and glucose (*t*-test, *P* < 0.005), and significantly lower plasma lactate (MWU, *P* < 0.001) and potassium (MWU, *P* < 0.001) than fish that became moribund (Fig. 4).

### qRT-PCR results

**Figure 8:**
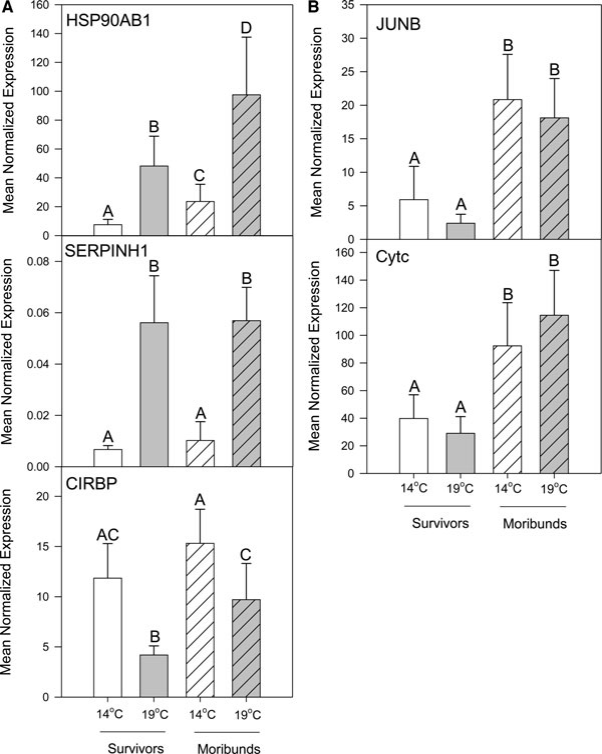
Differences in gene expression associated with (A) the temperature treatments (14°C, white bars; 19°C, gray bars) or (B) survival (Survivors, open bars; moribund fish, hatched bars) as determined by quantitative real-time PCR (qRT-PCR) to validate the microarray results. Different letters indicate statistically significant differences.

The five genes used to validate the microarray results showed the same directional change as determined by the microarrays (Fig. 8). For both survivors and moribund fish, HSP90AB1 (*F* = 120.7, *P* < 0.0001) and SERPINH1 (*F* = 269.3, *P* < 0.0001) had significantly higher mean normalized expression in fish held at 19°C compared with fish held at 14°C, and CIRBP (*F* = 71.3, *P* < 0.0001) had lower mean normalized expression in fish held at 19°C. These data provide some validation that the *P* < 0.001 array results were providing true positives, despite the poor level of *q*-value support. HSP90AB1 (*F* = 36.2, *P* < 0.0001) and CIRBP (*F* = 36.3, *P* < 0.0001) were also significantly affected by survival versus moribund status, as both were expressed at a higher level in moribund fish than survivors in the qRT-PCR analysis; however, these genes were not among the 751 most differentially expressed genes in the array analysis. Cytochrome C (cyt c [≍Cycs]; *F* = 92.9, *P* < 0.0001) and JUNB (*F* = 128.6, *P* < 0.0001) also had significantly higher mean normalized expression in the moribund group compared with survivors, as predicted from the microarray data.

## Discussion

### Temperature patterns

River temperatures reaching 19°C become stressful for migrating adult Fraser River sockeye salmon (Macdonald et al. 2000), but this temperature has not been historically considered acutely lethal (e.g., survival <24 h; Servizi and Jensen 1977). Nevertheless, migration for several days at ≥19°C results in increased en route mortality in at least some sockeye salmon populations (Martins et al. 2011). Additionally, chronic exposure to 19°C in a laboratory study resulted in increased mortality and evidence of a stress response in the blood of sockeye and pink salmon (Jeffries et al. 2012). Indeed, in the present study there was evidence of a thermal stress response in fish held at 19°C compared with fish held at 14°C in both the gene expression profiles and blood properties. Regardless of survival status, the genes HSP90AB1 and CIRBP, which are thermally responsive (Sonna et al. 2002), were transcriptionally modified because of the high temperature treatment in the present study. The directional changes in those genes were similar to the changes detected in thermally stressed arctic charr (*Salvelinus alpinus*; Quinn et al. 2011), and in wild sockeye salmon experiencing a 7–8°C increase in water temperature when transitioning from saltwater to freshwater (Evans et al. 2011). The upregulation of molecular chaperones in fish is a common response to elevated water temperatures. The collagen-specific endoplasmic reticulum resident chaperone SERPINH1, involved in collagen stabilization during stress (Krone et al. 1997), was upregulated at 19°C. In addition to the heat shock proteins HSP90AB1 and SERPINH1, the molecular chaperone PDIA4, which is involved in protein folding and is thermally responsive in fish (Logan and Somero 2010), was also upregulated at 19°C. The gene CIRBP is involved in RNA stabilization and has been associated with osmotic stress in fish (Pan et al. 2004; Evans 2010); however, it is also involved in a cold shock response in vertebrates (Sonna et al. 2002). These results show that CIRBP is thermally responsive in sockeye salmon over a 5°C temperature range. However, given that fish in the 19°C treatment also showed an increase in plasma chloride, suggesting a disturbance in osmoregulatory homeostasis and consistent with previous work (Jeffries et al. 2012), we cannot discern whether CIRBP was responding directly to temperature or to an osmotic perturbation. Additionally, there was an increase in plasma lactate and potassium in fish held at 19°C compared with those held at 14°C indicating that the 19°C treatment induced a stress response detectable in the blood properties.

Protein biosynthesis represents a significant portion of the cellular energy budget. In some eurythermal fish, protein biosynthesis may be upregulated to potentially compensate for higher rates of protein turnover at high temperatures (Logan and Somero 2010). However, the present study found that several genes involved in protein biosynthesis were downregulated at 19°C in sockeye salmon (CIRBP, hyperosmotic glycine rich protein, splicing factor, arginine/serine-rich 2, PRIC285, and CIP29). Additionally, genes involved in mRNA translation (elongation and initiation factors: EEF2, EIF3S6 [also EIF5 and EIF3H correlated with PC3] in survivors; EEF2L in moribunds) and various ribosomal proteins were downregulated in sockeye salmon held at 19°C (e.g., RPL7A and RPL3 correlated with PC3 in survivors; RPL31 and RPS25 in fish that became moribund). Many functional categories involved in protein biosynthesis were significantly enriched in both the survivors and moribund groups due to the temperature treatments. These results suggest that adult sockeye salmon may alter protein biosynthesis during periods of heat stress. Cellular energy expenditure may be significantly reduced by downregulating protein biosynthesis in energy stressed cells (Staples and Buck 2009); hence, a downregulation of nonessential protein biosynthesis may be a strategy to conserve finite energy stores during a temperature-induced increase in metabolic rate.

The upregulation of genes involved in immunity (e.g., CD74, H2-EB1, interferon regulatory factor 1 [IRF1], tumor necrosis factor alpha-induced protein 2 [TNFAIP2], all correlated with PC3; Ig kappa chain V-IV region B17 precursor [no gene symbol available] in survivors; MGC84465 in moribund fish) in sockeye salmon held at 19°C may result from temperature stress, but could also be stimulated by the enhanced virulence of many salmon diseases at higher temperatures. Pacific salmon are exposed to a wide variety of pathogens and parasites when they transition back to freshwater during spawning migrations, many of which have temperature-dependant progressions (Rucker et al. 1954). ErmineJ ROC analysis of PC3 confirmed that functional categories relating to an immune response were significantly affected by the high water temperature treatment in survivors. Indeed, many of the genes most correlated with PC3 are involved in immunity, rather than a thermal stress response as determined by the supervised analysis approach. Fraser River sockeye salmon are often affected by diseases caused by the bacteria *Flexibactor columnaris*, the kidney parasite *Parvicapsula minibicornis,* and the fungus *Saprolegnia* sp., which all progress faster at higher temperatures (Servizi and Jensen 1977; Crossin et al. 2008).

### Mortality patterns

The most significantly upregulated gene in sockeye salmon that became moribund compared with survivors was ODC1, which is involved in polyamine synthesis and has been linked to cellular apoptosis (Pignatti et al. 2004). Accumulation of polyamines in the cell may increase the permeability of mitochondrial membranes, which results in cyt c and other proteins being released into the cytoplasm and initiates the postmitochondrial phase of apoptosis (Pignatti et al. 2004). Polyamines are also involved in the phospho-rylation of MAPK, which leads to the upregulation of several transcription factors (Bachrach et al. 2001), like JUNB, that may be involved in apoptosis. Along with the upregulation of ODC1, we detected an upregulation of cyt c and JUNB, both of which are linked to cell apoptosis. The apparent role of ODC1 in cellular apoptosis in dying sockeye salmon is an interesting discovery and warrants further investigation.

The extracellular signal(s) that triggered the upregulation of ODC1 in gill tissue in moribund sockeye salmon is unknown. However, there is evidence that ODC1 is upregulated during periods of hypoosmotic stress (Watts et al. 1996; Lockwood and Somero 2011). Interestingly, sockeye salmon plasma ions decrease throughout their adult freshwater residency (Shrimpton et al. 2005) and mortality is preceded by drastic decreases in plasma chloride, sodium, and osmolality that begin days before the fish dies (Jeffries et al. 2011). We detected lower levels of plasma chloride and sodium in moribund fish at both temperatures, which could effectively create a hypoosmotic stress for cells and potentially lead to the increase in ODC1. Indeed, ODC1 was one of the genes most strongly correlated with plasma chloride levels. There is also evidence that cellular ODC1 activity may be stimulated by cortisol (Wu et al. 2000), a hormone that increases in the plasma of sockeye salmon at death (Hruska et al. 2010; Jeffries et al. 2011). Therefore, the osmoregulatory failure and spike in plasma cortisol that occur in moribund sockeye salmon could be linked with the upregulation of ODC1 in gill tissue. To our knowledge, this is the first evidence of this potential relationship between apoptosis and ODC1 upregulation in dying Pacific salmon and in any other dying semelparous animal.

There was increased expression of transcription factors, in addition to JUNB, in fish that became moribund. Nuclear protein 1 (NUPR1), which is a stress-responsive transcription factor involved in a wide variety of cell functions (Chowdhury et al. 2009), including cell growth, was upregulated. An increase in NUPR1 may also be related to or correlated with elevated cortisol levels in a generalized fish stress response (Momoda et al. 2007). Because cortisol levels are known to be elevated in moribund Pacific salmon (Hruska et al. 2010; Jeffries et al. 2011), a potential relationship between NUPR1 and cortisol is possible in moribund sockeye salmon. The transcription factor CCAAT/enhancer binding protein beta (CEBPB), which has a role in cell proliferation (Lekstrom–Himes and Xanthopoulos 1998), was also upregulated in moribund salmon. However, CEBPB is also involved in an inflammatory response (Lekstrom-Himes and Xanthopoulos 1998) and therefore its upregulation may also be related to processes involved in immune function.

Adult sockeye salmon become progressively immuno-suppressed throughout their freshwater spawning migration, and disease has been suggested as the ultimate cause of death in adult Pacific salmon (Gilhousen 1990). Several genes involved in the immune and inflammatory response (e.g., chemokine C-C motif ligand 19 [CCL19], allograft inflammatory factor 1 [AIF 1], and complement factor D [CFD]) were downregulated in moribund sockeye salmon, consistent with immunosuppression. Indeed, ErmineJ ROC analysis indicated that the functional categories “defense response” and “response to wounding” were significantly enriched in PC1. Additionally, many of the genes strongly correlated with plasma chloride levels, used as a proxy for survival status, were associated with an immune response (e.g., CD74, H2-EB1, ICLP2), indicating the complex relationship between plasma chloride, osmoregulatory ability, immunosuppression, and other molecular processes that occur in gill tissue as fish die. Many contigs of the antigen processing and presenting gene major H2-EB1, and the associated CD74, were also downregulated in moribund sockeye salmon. Additionally, the transcription activator IRF1, involved in regulating MHC class I and II related genes (Young et al. 2008), was downregulated in moribund fish. MHC II genes are involved in the humoral immune response generally associated with extracellular pathogens such as bacteria and parasites. By downregulating humoral immunity during senescence, bacterial infections like those caused by *F. columnaris*, common in Fraser River sockeye salmon and believed to cause significant mortality in some Pacific salmon populations (Servizi and Jensen 1977), could become more virulent.

## Conclusions

The semelparous life history of Pacific salmon allows for only one opportunity to migrate to spawning grounds and successfully spawn. Spawning migrations during warm water periods may lead to increased levels of premature mortality for sockeye salmon, which results in a lifetime fitness of zero for those individuals. The present study is the first to demonstrate the effects of water temperature and mortality on the cellular-level physiology of wild-caught Pacific salmon held under controlled conditions. We show that the gill transcriptome of sockeye salmon held for seven days at 19°C showed increase in heat shock and immune responses, and a decrease in expression of genes involved in protein bio-synthesis compared with salmon held at 14°C. These patterns were common between the two populations. There were also indications of individual variability in adaptive responses to thermal stress that may have been associated with survival; the fish that did not respond similarly to other fish in the high temperature treatment, but survived seven days, were dead by day 9, potentially because of their lack of an appropriate cellular response to temperature stress. This is an interesting observation that warrants further investigation. Our data also suggest that Pacific salmon may be increasingly affected by temperature-dependent diseases when migrating during periods of elevated (but not acutely lethal) water temperatures, which can lead to premature mortality. Despite the fact that neither population nor sex were identified as strong factors in the gene expression profiles in the present study, recent work has shown the importance of considering population- (Eliason et al. 2011) and sex-specific (Clark et al. 2009; Sandblom et al. 2009; Jeffries et al. 2012) differences in Fraser River sockeye salmon populations. Therefore, future studies should specifically examine the role of population- and sex-specific differences in thermal tolerance at the cellular level. This study also presents some of the first data showing the gene expression changes associated with premature mortality, which may or may not parallel final senescence, and suggests that ODC1-mediated processes are involved in cell death in dying Pacific salmon. Genetic and biochemical processes associated with mortality may be useful in developing assays for understanding causes of premature mortality and predicting the fate of adult Pacific salmon throughout the migration and upon arrival at spawning grounds.

## Supporting Information

**Table S1.** Annotation information for genes that had different levels of expression at *P* < 0.001 (Mann–Whitney U tests) between temperature treatment groups for (A) survivors, (B) moribund fish, or (C) between survivors andmoribund fish.
